# Dehydroalanine and dehydrobutyrine in aging and cataractous lenses reveal site-specific consequences of spontaneous protein degradation

**DOI:** 10.3389/fopht.2023.1241001

**Published:** 2023-10-26

**Authors:** Jessica Paredes, Zhen Wang, Purvi Patel, Kristie L. Rose, Kevin L. Schey

**Affiliations:** ^1^ Department of Chemistry, Vanderbilt University, Nashville, TN, United States; ^2^ Department of Biochemistry, Vanderbilt University, Nashville, TN, United States; ^3^ Mass Spectrometry Research Center, Vanderbilt University, Nashville, TN, United States

**Keywords:** post-translational modifications, proteomics, lens, cataracts, aging

## Abstract

**Introduction:**

Protein post-translational modifications (PTMs) have been associated with aging and age-related diseases. PTMs are particularly impactful in long-lived proteins, such as those found in the ocular lens, because they accumulate with age. Two PTMs that lead to protein-protein crosslinks in aged and cataractous lenses are dehydroalanine (DHA) and dehydrobutyrine (DHB); formed from cysteine/serine and threonine residues, respectively. The purpose of this study was to quantitate DHA and DHB in human lens proteins as a function of age and cataract status.

**Methods:**

Human lenses of various ages were divided into five donor groups: transparent lenses (18–22-year-old, 48–64-year-old, and 70–93-year-old) and cataractous human lenses of two age groups (48–64-year-old lenses, and 70–93-year-old lenses) and were subjected to proteomic analysis. Relative DHA and DHB peptide levels were quantified and compared to their non-modified peptide counterparts.

**Results:**

For most lens proteins containing DHA or DHB, higher amounts of DHA- and DHB-modified peptides were detected in aged and cataractous lenses. DHA-containing peptides were classified into three groups based on abundance changes with age and cataract: those that (1) increased only in age-related nuclear cataract (ARNC), (2) increased in aged and cataractous lenses, and (3) decreased in aged lenses and ARNC. There was no indication that DHA or DHB levels were dependent on lens region. In most donor groups, proteins with DHA and DHB were more likely to be found among urea-insoluble proteins rather than among water- or urea-soluble proteins.

**Discussion:**

DHA and DHB formation may induce structural effects that make proteins less soluble in water that leads to age-related protein insolubility and possibly aggregation and light scattering.

## 1 Introduction

The function of the ocular lens depends on the stability of long-lived proteins due to the almost non-existent protein turnover in mature lens fiber cells (1–4). However, the vulnerability of long-lived proteins to spontaneous degradation has been implicated in the formation of high-molecular weight crosslinks and protein aggregation in the lens (4–7). Highly studied age-related post-translational modifications (PTMs) such as deamidation (8, 9), oxidation (10–13), and crosslinking (14–16) have been identified in lens proteins and linked to age-related diseases in other tissues (15, 17). Proteins in the lens are exposed to reactive oxygen species during aging and some oxidized products, such as protein disulfides, can be reversed by protective mechanisms (18–20). Oxidative stress repair biomolecules in the lens, such as glutathione (GSH), and small heat-shock proteins, such as α-crystallins, are subject to age-related decline or modification (21–24). Increased oxidation with age can result in irreversible oxidation products that limit the ability of protective biomolecules to maintain lens transparency (25, 26). Over time, oxidative damage accumulates in the lens, which leads to age-related nuclear cataracts (ARNC) (16, 27). Opacification of the nucleus of the lens in ARNC has been associated with an increase of high-molecular weight crosslinks, protein-protein aggregation, and a reduction of protein-solubility (28).

**Figure 1:**
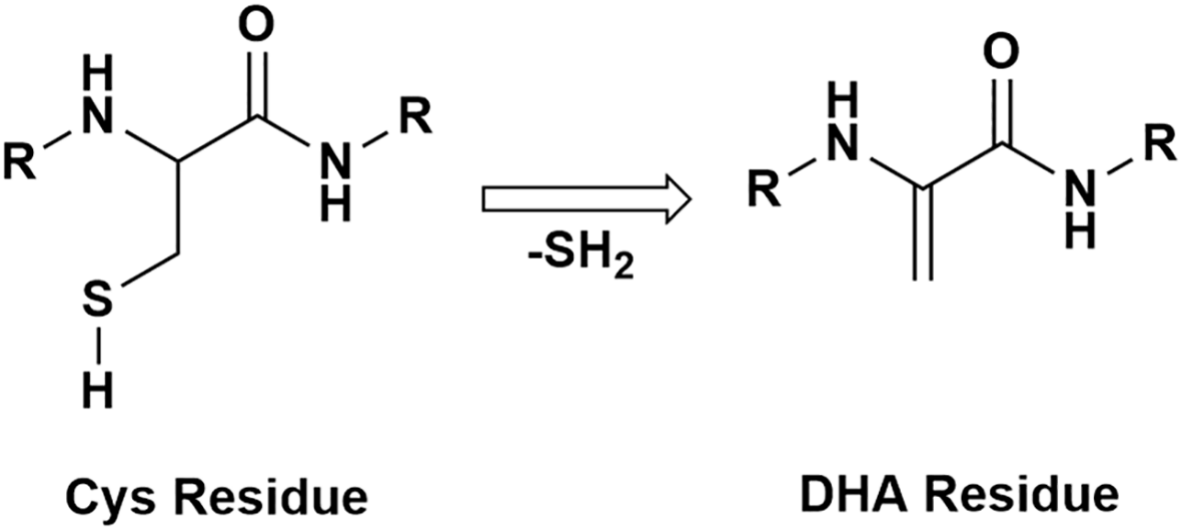
A schematic of a protein with a cysteine residue undergoing β-elimination and forming DHA.

Irreversible PTMs can result in protein-protein crosslinking and aggregation. Reactive intermediates that lead to irreversible crosslinking are dehydroalanine (DHA) and dehydrobutyrine (DHB) (29). The formation of DHA and DHB residues in proteins is caused by a β-elimination reaction occurring on cysteine, serine, phosphoserine residues for DHA and on phosphothreonine, or threonine residues for DHB. A scheme for DHA formation is shown in **Figure 1**. DHA has been identified in long-lived proteins in lens tissue (30) and has been hypothesized to form via both nonenzymatic and enzymatic mechanisms (31, 32). After DHA or DHB form, these intermediates can react with nucleophilic residues in proteins such as cysteine to form lanthionine, histidine to form histodinoalanine, and lysine to form lysinoalanine (33). These reaction products have been identified in significantly higher levels in cataractous lenses compared to age-matched transparent lenses (33, 34). DHA can also react with cysteine on GSH, and DHA-GSH crosslinks on essential proteins for lens transparency have been associated with aging and cataracts (25). Although DHA-mediated crosslinks have been identified, the stability, abundance, and effects of DHA and DHB modifications have not been examined. In this study, we quantified the abundances of DHA and DHB in five groups of transparent and cataractous human lenses across the age ranges 18-22-years-old, 48-64-years-old, 48-64-years-old with cataracts, 70-93-years-old, and 70-93-years-old with cataracts. Our results suggest that DHA and DHB formation can lead to insolubilization of multiple lens proteins and is often elevated in cataract lenses.

## 2 Materials and methods

The methods of preparing, measuring, and analyzing data were conducted as outlined in Wang and Schey in 2018 (25).

### 2.1 Lens samples

Senior donors with and without advanced stages of ARNC were received as a gift from Dr. Donita Garland. Lenses older than 70 years old were selected for proteomic analysis and data collected were added to the dataset from Wang and Schey (25). An image of each lens in this older group, before dissection, can be found in **Supplementary Figure 1**. Meta data for each lens can be found in **Table 1**. Lenses analyzed by Wang and Schey (25) were from donors in the following groups: ages 18-22-years-old with no cataract history, ages 48-64-years-old with no cataract history, and ages 48-64-years-old with cataract (25). These lenses were obtained from NDRI (Philadelphia, PA). Each group had 4 lenses except for 70-93-years-old lenses, which was composed of three lenses. Lenses were excluded if lenses were deformed or were badly dissected. Two lenses with ARNC, aged 70 and 75, had both nuclear cataracts and posterior subcortical cataracts.

**Table 1:** Age, cataract status, and sex of lenses analyzed.

Age	Grouping	Cataract Status	Sex
18	18-22	None	Female
19	18-22	None	Male
21	18-22	None	Male
22	18-22	None	Female
48	48-64	None	Male
53	48-64	None	Male
53	48-64	None	Male
56	48-64	None	Male
50	48-64 & cat	Nuclear Cataract	Male
57	48-64 & cat	Nuclear Cataract	Male
58	48-64 & cat	Nuclear Cataract	Male
64	48-64 & cat	Nuclear Cataract	Male
78	70-93	None	*
82	70-93	None	*
93	70-93	None	*
70	70-93 & cat	Nuclear Cataract	*
72	70-93 & cat	Nuclear Cataract; Posterior Subcortical Cataract	Male
75	70-93 & cat	Nuclear Cataract; Posterior Subcortical Cataract	Male
80	70-93 & cat	Nuclear Cataract	Male

*Sex information unavailable.

### 2.2 Lens dissection

Anterior and posterior regions of the lens were removed by sectioning equatorially at 30 µm thickness using a Lecia CM3050 S Research Cryostat (Leica Biosystems, Buffalo Grove, IL, USA) and about 1 mm from the anterior pole and 1.5 mm from the posterior pole were removed. Lens cortex (C), outer nucleus (ON), and inner nucleus (IN) regions were dissected from the remaining uncut lens based on radial distance from the lens center using surgical trephines. The IN was defined as the lens region from the lens center to a radial distance of 4.5 mm. The ON was defined as the region between a radial distance of 4.5 to 6 mm. Lens tissue greater than a radial distance of 6 mm was defined as the cortex.

### 2.3 Lens homogenization and fractionation

Lens tissue was manually homogenized in homogenization buffer comprised of 25 mM Tris, 150 mM NaCl, and 5 mM EDTA and centrifuged at 20,000 g for 30 minutes. The supernatant was isolated and defined as the water-soluble fraction (WSF). The pellet was solubilized in 8M urea, 25 mM Tris, 150 mM NaCl, and 5 mM EDTA and centrifuged at 20,000 g for 30 minutes. The supernatant was isolated, and this fraction was defined as the urea-soluble fraction (USF). The remaining pellet was suspended in water and proteins from this fraction were defined as the urea-insoluble fraction (UIF).

### 2.4 Sample preparation for liquid chromatography and tandem mass spectrometry

Proteins from each region and fraction (WSF-IN, WSF-ON, WSF-C, USF-IN, USF-ON, USF-C, UIF-IN, UIF-ON, and UIF-C) were quantified via a bicinchoninic acid (BCA) assay. 50 µg from each region and fraction were aliquoted for proteomic analysis. Sample proteins were reduced via incubation in 10 mM DTT at 56°C for one hour. Proteins were alkylated with iodoacetamide, added to a final concentration of 55 mM, and incubated in the dark at room temperature for 45 mins. WSF and USF fractions were precipitated by methanol-chloroform as described by Wessel et al. (35). UIF fractions in water were spun down at 20,000 g for 20 minutes in 4°C and the supernatant was removed. Precipitated WSF, USF, and pelleted UIF fractions of each region were suspended in 10 µL acetonitrile and diluted with 90 µL of 50 mM Tris. Proteins were digested by the addition of trypsin at a 1:50 trypsin to protein ratio. Samples were dried via SpeedVac and re-solubilized in 0.1% formic acid. Samples were cleaned with stop and go extraction (STAGE) tips and analyzed with liquid chromatography tandem mass spectrometry (LC-MS/MS).

### 2.5 Liquid chromatography and tandem mass spectrometry

Tryptic peptides corresponding to 0.5 µg of total protein were separated on a one-dimensional fused silica capillary column (100 µm x 20 cm). This column was packed with C_18_ Phenomenex Jupiter resin (3 µm mean particle size, 300 Å pore size) and coupled with an Dionex Ultimate 3000 nanoLC (Thermo Scientific, San Jose, CA). A 90-minute gradient was performed, consisting of: 1-68 minutes at 2-38% acetonitrile (ACN) in 0.1% formic acid, 68-74 minutes at 38-95% ACN in 0.1% formic acid, 74-75 minutes at 95% ACN in 0.1% formic acid, 76-76 minutes at 95-2% ACN in 0.1% formic acid and 76-85 at 2% ACN in 0.1% formic acid. The mobile phase was balanced with 0.1% formic acid. The eluate was directly infused into a Q Exactive Plus instrument (Thermo Scientific, San Jose, CA) equipped with a nanoelectrospray ionization source. The data-dependent acquisition method consisted of MS1 acquisition (R=70,000) using an MS AGC target value of 3e6, followed by up to 15 MS/MS scans (R=17,500) of the most abundant ions detected in the preceding MS scan. The MS2 AGC target value was set to 1e5, with a maximum ion time of 100 milliseconds, and intensity threshold of 3e4. HCD collision energy was set to 27 NCE, and dynamic exclusion was set to 10 seconds, and peptide match and isotope exclusion were enabled.

### 2.6 MaxQuant search

For identification of DHA and DHB modification sites, the raw data were processed and searched against a concatenated forward and reversed (decoy) human Swissprot (Oct 2022) database. The data were searched with MaxQuant version 2.1.4.0. The false discovery date (FDR) was set to 1%. Differential modifications included carbamidomethylation of cysteine, oxidation of methionine, Gln conversion to pyro Glu, DHA modifications on cysteine (-33.9877 Da) and serine (-18.0106 Da), and DHB modification on threonine (-18.0106 Da). All modified peptides sequences were manually verified by inspection of peptide tandem mass spectra with 5 ppm mass accuracy for parent masses and 10 ppm for product ions.

### 2.7 Data analysis

Selected ion chromatograms of modified and non-modified peptides were extracted using the Qual Browser tool in Xcalibur software (Thermo Scientific) using a 10 ppm mass tolerance. Peak areas of DHA modified peptides and non-modified peptides were calculated for all charge states and the most intense three isotopes were summed. The relative level of modification was defined as the ratio of the peak area of modified peptide to the peak area of the non-modified summed with the modified peptide and multiplied by 100. The results are presented as mean +/- standard deviation of each modified peptide abundance in each solubility fraction and each region of four different lenses in each group. The data were analyzed in R 4.2.2. A factor analysis of mixed data (FAMD) was done with FactoMineR. Samples without peak areas for modified peptides were not used in further statistical analysis (36). Quantile normalization was done to account for biological variability (37). To determine relationships between age groups within lenses of the same solubility fraction and region, an analysis of variance (ANOVA) was used followed by a *post-hoc* Tukey test. Results were considered statistically significant when p < 0.05.

## 3 Results

### 3.1 Aging and cataracts influence the lens proteome

**Figure 2:**
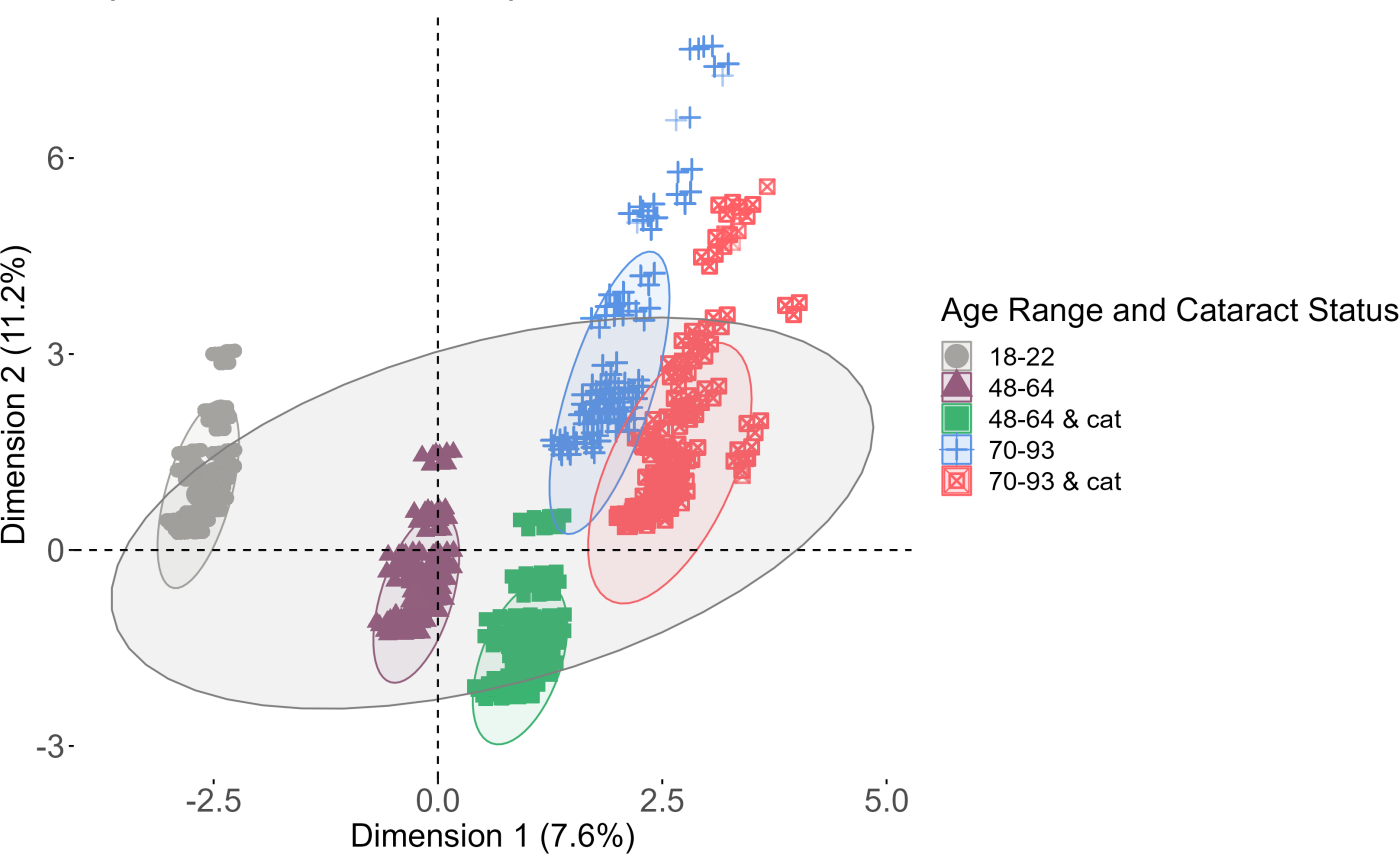
Factor analysis of mixed data (FAMD) analysis. Each ellipse represents a 95% confidence level among each group of data (lenses 18-22, 48-64, 46-64 with cataracts, 70-93, and 70-93 with cataracts). The largest ellipse is a confidence level among the entire data set.

Global changes in the lens proteome were analyzed using a data dimensionality reduction method, factor analysis of mixed data (FAMD), to assess the variables that most contributed to variance in the data. The results of the FAMD analysis are shown in **Figure 2**. The six variables compared in the FAMD included: solubility, region, protein presence, protein sequence coverage, age, and age range and cataract status of each peptide identified by MaxQuant. Age and protein sequence coverage were continuous data while the others were categorical data. The variables that contributed to Dimension 1 were age and age range and cataract status whereas Dimension 2 was most affected by the protein identified and its corresponding sequence coverage. Data for each lens group as well as the complete data were grouped by an ellipse representing a 95% confidence interval.

Each group of data has the same orientation against both axes, which suggests that the data have similar distributions. The datasets clearly segregate by age. Healthy lenses compared to cataractous lenses have large overlaps, suggesting that proteins were more likely to be affected by age than by cataract status. The overlap among middle-aged and senior lenses suggests that these data have more in common with one another than with younger lenses, which has no overlap with any other group. The total variance extracted from both dimensions of the data was 18.8%, suggesting that the data is well-represented by both dimensions. Overall, most of the data in each group is within a 95% confidence interval of the mean of the entire data set, which indicates that previously acquired data can be compared to recently acquired data.

### 3.2 DHA and DHB abundances

**Figure 3:**
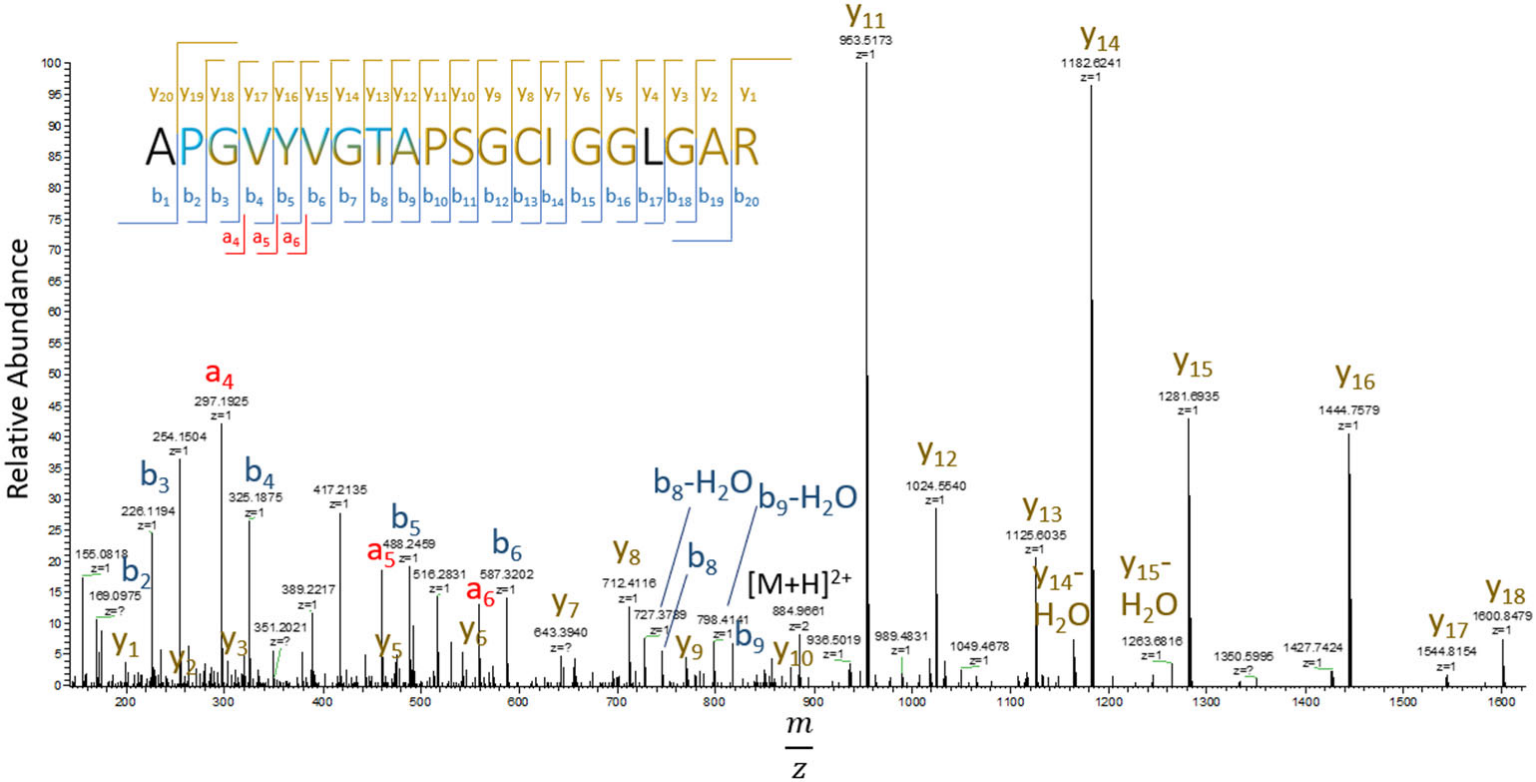
Tandem mass spectrum of residues 53-72 (APGVYVGTAPSGCIGGLGAR) of BFSP2 (phakinin). DHA is formed from hydrogen sulfide loss from cysteine with a mass shift of -33.9877 Da. b-ions are labeled in blue, y-ions yellow, and a-ions are red. Letters in black refer to residues not identified by b, y, and a ions.

Previous identification of DHA in lens proteins has been limited to Ser59 on αA-crystallin (30), which was not detected in the current study. To the best of our knowledge, the 24 sites of DHA and DHB identified in our study are novel. Manual inspection of DHA containing peptide MS/MS spectra revealed that DHA formation was more commonly observed at cysteine residues compared to DHA or DHB formation at serine or threonine residues. DHA and DHB formed from water loss were often the result of artifactual in-source decay during analysis, i.e. water loss from glutamic and aspartic acid misidentified as DHA or DHB, or a potential loss of phosphoric acid from phosphoserine or phosphothreonine residues. To avoid analysis of artifactual DHA or DHB formation, only peptides that displayed a shift in retention time between modified and non-modified sequences were selected for quantitation. Among the entire dataset, 20 sites were found with cysteine-based DHA, 2 sites showed DHB from water loss, and 1 DHA site from water loss (**Table 2**). An example of a DHA-modified peptide tandem mass spectrum is shown in **Figure 3**. The masses of y_7_ and y_8_ ions define the site of DHA as occurring on residues Cys65 in phakinin (BFSP2). Among the detected peptides, the relative levels of modification on 13 unique peptides were abundant enough in most samples to be quantified.

**Table 2:** Identified sites of DHA and DHB on lens proteins.

Protein: Peptide Residues	Modified Residue	Peptide with Modification
**αA-crystallin:** 118-145	C131, C142	YRLPSNVDQSALS**C(ALK)**SLSADG**M(OX)**LTF**C(DHA)**GPK
**αB-crystallin:** 22-56	S35	LFDQFFGEHLLE**S(DHA)**DLFPTSTSLSPFYLRPPSFLR
**αB-crystallin:** 164-175	T170	EEKPAV**T(DHB)**AAPK
**βA3-crystallin:** 46-58	C52	**M(OX)**EFTSS**C(DHA)**PNVSERSFDNVR
**βA3-crystallin:** 65-95	C82	SLKVESGAWIGYEHTSF**C(DHA)**GQQFILERGEYPR
**βA3-crystallin:** 126-137	T127	**M(OX)T(DHB)**IFEKENFIGR
**βA3-crystallin:** 163-177	C170	IQSGAWV**C(DHA)**YQYPGYR
**βA4-crystallin:** 2-7	C5	**T(ACE)**LQ**C(DHA)**TK
**βA4-crystallin:** 26-45	C33	RHEFTAE**C(DHA)**PSVLELGFETVR
**βA4-crystallin:** 159-177	C166	GFQYVLE**C(DHA)**DHHSGDYK
**βB1-crystallin:** 73-92	C79	RAEFSGE**C(DHA)**SNLADRGFDRVR
**βB2-crystallin:** 49-81	C67	AGSVLVQAGPWVGYEQAN**C(DHA)**KGEQFVFEKGEYPR
**γB-crystallin:** 16-32	C19, C23	SYE**C(DHA)**TTD**C(ALK)**PNLQPYFSR
**γC-crystallin:** 16-32	C23	SLHVLEG**C(DHA)**WVLYELPNYR
**γD-crystallin:** 16-38	C19	HYE**C(DHA)**SSDHPNLQPYLSR
**γD-crystallin:** 102-117	C109, C111	EDYRGQMIEFTED**C(DHA)**S**C(ALK)**LQDRFR
**γS-crystallin:** 20-36	C23, C25, C27	YD**C(ALK)**D**C(ALK)**D**C(DHA)**ADFHTYLSR
**BFSP1:** 255-276	C259	SAHE**C(DHA)**YDDEIQLYNEQIETLRK
**BFSP2:** 53-72	C65	APGVYVGTAPSG**C(DHA)**IGGLGAR
**BFSP2:** 90-121	C114	SSGLATVPAPGLERDHGAVEDLGG**C(DHA)**LVEYMAK
**BFSP2:** 157-173	C161	ASWASS**C(DHA)**QQVGEAVLENAR
**BFSP2:** 251-276	C255	QLAG**C(DHA)**ELEQMDAPIGTGLDDILETIR
**BFSP2:** 318-339	C326	VELHNTS**C(DHA)**QVQSLQAETESLR
**CBR1:** 119-134	C122	DV**C(DHA)**TELLPLIKPQGR

*ALK refers to alkylated and a mass shift of 57.0215. OX refers to oxidized and a mass shift of 15.9949. ACE refers to acetylated and a mass shift of 42.0106. C(DHA) refers to a mass shift of -33.9877. S(DHA) and T(DHB) refer to a mass shift of -18.0106. BFSP1 and BFSP2 refer to filensin and phakinin, respectively.

### 3.3 DHA-modified peptides associated with only ARNC

**Figure 4:**
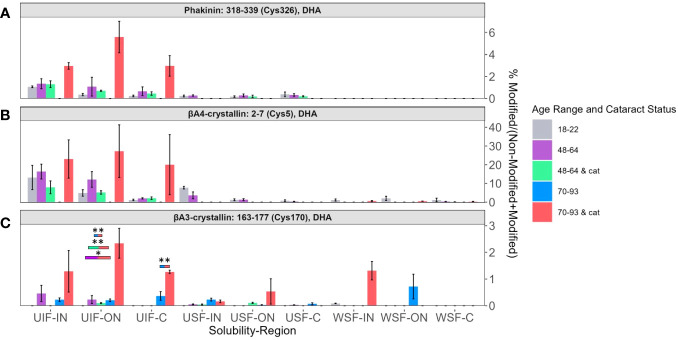
DHA containing peptides abundant in age-related nuclear cataract. Relative levels of DHA are shown for **(A)** BFSP2 (Phakinin) Cys326, **(B)** βA4-crystallin Cys5, and **(C)** βA3-crystallin Cys170 among each fractionated region, solubility group, and donor group. WSF-IN, WSF-ON, and WSF-C refers to the water-soluble inner nucleus, outer nucleus, and cortex. USF-IN, USF-ON, and USF-C refer to the urea-soluble inner nucleus, outer nucleus, and cortex. UIF-IN, UIF-ON, and UIF-C refer to the urea-insoluble inner nucleus, outer nucleus, and cortex. Boxes above each result are colored to show which groups are statistically significantly (p<0.05) different. The boxes are colored according to the legend to indicate which bars are compared and show significant abundance differences. Boxes outlined in black represent comparisons where at least one lens group has cataracts; boxes without outlines represent comparisons where neither lens groups have cataracts. An asterisk (*) represents a p-value less than 0.05; ** represents a p-value less than 0.01.

DHA levels among each solubility fraction and lens region for BFSP2 Cys326 (**Figure 4A**), βA4-crystallin Cys5 (**Figure 4B**), and βA3-crystallin Cys170 (**Figure 4C**) were compared. DHA levels that were significantly different between different age groups and cataract status were denoted with color coded boxes above the bars being compared. Outlined boxes represent comparisons where one age group has cataracts. DHA levels were highest in the UIF for most age groups independent of region or cataract status for all three modified proteins. The relatively high abundance of DHA modified peptides in all UIF samples is noteworthy because non-modified crystallins are water soluble and non-modified BFSP2, being a cytoskeletal protein, is more likely to be identified in the USF.

In **Figure 4**, DHA abundance of BFSP2 Cys326, βA4-crystallin Cys5, and βA3-crystallin Cys170 were highest among senior donors with cataracts (pink bars) in all regions of the UIF. Transparent senior lenses (blue bars) displayed lower DHA levels when detected. Although not all DHA abundance differences between age groups in the UIF were statistically significant due to high variability among donors and a small sample size, a similar trend was observed for these three different proteins.

DHA levels on BFSP2 Cys326 and βA4-crystallin Cys5 in the UIF significantly increased from the C region to the IN region in lenses within each group: 18–22-year-old in BFSP2 Cys326 (**Figure 4A**), and 48–64-year-old with and without cataracts in βA4-crystallin Cys5 (**Figure 4B**). These results suggest a regional trend toward higher DHA content in older fiber cells in the UIF, which are the regions of the lens most impacted by ARNC. However, no trend could be identified when comparing younger, middle-aged, and senior lenses within each region in the UIF. The abundance of DHA-containing peptides did not show a strong association with aging even though a slight regional trend was detected.

βA3-crystallin Cys170 (**Figure 4C**) showed relatively high levels of DHA in senior cataractous lenses in the WSF-IN but not in the WSF-ON, where high levels of DHA were seen in transparent senior lenses. DHA-modified peptides of βB1-crystallin Cys79 in **Supplementary Figure 2** displayed similar trends where high levels of DHA were observed in senior lenses with cataracts in the WSF-IN and senior lenses without cataracts were highest in ON and C regions of the WSF. The highest levels of DHA on βB1-crystallin Cys79 were seen in the UIF. Surprisingly, relatively high levels of DHA were observed in the UIF compared to the WSF from young lenses suggesting a shift from a highly water-soluble protein to an insoluble protein upon DHA formation.

### 3.4 DHA-modified peptides associated with aging and ARNC

**Figure 5:**
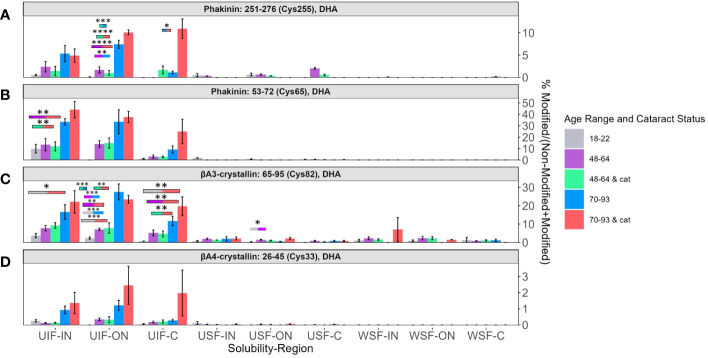
Relative abundance of DHA containing peptides associated with aging. Relative levels of DHA are shown for **(A)** BFSP2 (Phakinin) Cys255, **(B)** BFSP2 Cys65, **(C)** βA3-crystallin Cys82, and **(D)** βA4-crystallin Cys33. Boxes above each result are colored to show which groups are statistically significantly (p<0.05) different. Boxes outlined in black represent comparisons where at least one lens group has cataracts; boxes without outlines represent comparisons where neither lens groups have cataracts. An asterisk (*) represents a p-value less than 0.05; ** represents a p-value less than 0.01; *** represents a p-value less than 0.001; and **** represents a p-value less than 0.0001.

Four DHA sites whose abundances were strongly associated with lens age are BFSP2 Cys255 (**Figure 5A**), BFSP2 Cys65 (**Figure 5B**), βA3-crystallin Cys82 (**Figure 5C**), and βA4-crystallin Cys33 (**Figure 5D**). All four sites showed a high abundance of DHA in the UIF in all three regions of the lens. In each region of the UIF, DHA levels increased from younger to middle-aged to senior lenses. The other solubility fractions did not show any discernable trend. In the UIF, transparent senior lenses and senior lenses with cataracts (blue and pink bars respectively) showed greater differences in the C region than in the ON and IN regions.

In all four peptides, the range of levels of DHA between transparent and cataractous lenses in the IN and ON overlap. There is not a significant difference among these senior lenses. However, in BFSP2 Cys 255 in **Figure 5A**, a significant difference of DHA levels between transparent and cataractous senior lenses was seen (pink and blue bars, respectively). Similarly, significant regional differences between the IN region and C region in senior transparent lenses (blue bars) and regional differences in middle aged transparent and cataractous lenses between the ON region and C region (purple and green bars respectively) were seen in DHA levels in BFSP2 Cys65 in **Figure 5B**.

### 3.5 DHA-modified peptides not associated with aging or ARNC

**Figure 6:**
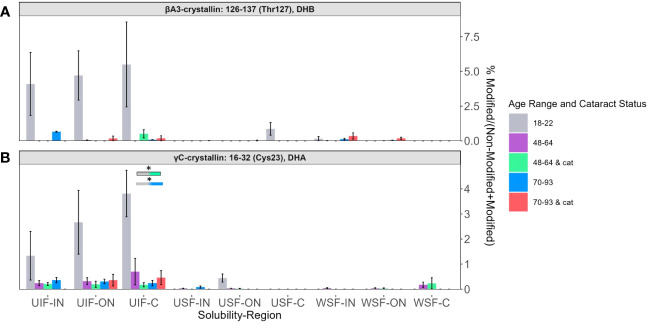
DHB and DHA containing peptides abundant in young lenses. Relative levels of DHB are shown for **(A)** βA3-crystallin Thr127 and of DHA for **(B)** γC-crystallin Cys23. Boxes above each result are colored to show which groups are statistically significantly (p<0.05) different. Boxes outlined in black represent comparisons where at least one lens group has cataracts; boxes without outlines represent comparisons where neither lens groups have cataracts. An asterisk (*) represents a p-value less than 0.05.

Relative abundances of two peptides: βA3-crystallin T127 containing DHB (**Figure 6A**) and γC-crystallin Cys23 containing DHA (**Figure 6B**) showed, again, that the highest levels of modification were detected among the UIF samples. In contrast, to the peptides discuss above, DHA and DHB were more likely to be detected in 18-22-year-old lenses for these two peptides. The relative lack of these modifications in lenses from older subjects suggests that DHA and DHB at these sites may reacting with lens nucleophiles over time.

DHA in filensin (BFSP1) Cys259 was more likely to be present in younger lenses (**Supplementary Figure 3**) like the trends seen in **Figure 6**. In fact, DHA was not detected in senior lenses with or without cataracts in most regions and fractions. DHA in BFSP1 Cys259 also exhibited a solubility shift from the expected presence in the USF to predominant present in the UIF. Interestingly, transparent 48-64-year-old lenses showed relative high abundance in the WSF-C compared to all other age groups. Although it is unclear as to why such changes in hydrophobicity would occur, these results provide additional evidence that that DHA may be involved in structural changes that affect the overall protein solubility. In fact, DHA was not detected in senior lenses with or without cataracts in most regions and fractions. The results from this site also suggest that DHA and DHB may be participating in other reactions.

Although most detected DHA modified peptides were abundant in the UIF, there were notable exceptions. The DHA site that displayed unique changes in solubility was αA-crystallin, residues Cys131 and Cys142, which is typically a water-soluble protein. DHA in αA-crystallin Cys131 and Cys142 (**Supplementary Figure 4**) showed an age-related trend in the ON and C regions of the UIF but not in the IN region of UIF, which is the region most impacted by aging and cataracts. Additionally, DHA levels among senior cataractous lenses were only high among the UIF whereas age-related trends could be seen in the USF except for senior cataractous lenses. Interestingly, DHB in αB-crystallin T170 did not exhibit a solubility shift (**Supplementary Figure 5**), unlike most of the other sites discussed above, and no discernable trends were identified.

## 4 Discussion

Twenty-four new sites of DHA and DHB were identified on thirteen human lens proteins. Of these sites, thirteen were quantified as a function of age, solubility, and lens region. For most proteins, the presence of DHA or DHB resulted in a shift of the protein to the insoluble fraction. In addition, three general trends were observed in aged and cataractous lenses: (1) DHA/DHB abundance increased with ARNC but not age, (2) DHA/DHB abundance increased with both age and ARNC, and (3) DHA/DHB abundance had no relationship with aging and ARNC.

DHA and DHB modifications were identified in many of the same protein residues and tertiary structures as previously reported to contain DHA or DHB-mediated crosslinks (25) and cysteine oxidation (13, 17). The correlation of DHA to these previously published sites reaffirms that there may be residues, such as cysteine, that may be ‘hotspots’ for spontaneous degradation (38). The presence of DHA and DHB in long-lived lens proteins suggests a stability not previously considered. Although subjects of nucleophilic attack by lysine, cysteine, and histidine residues, our results demonstrate that DHA and DHB can be relatively stable, and their reactivity may depend on surface exposure and/or proximity to nucleophilic residues.

Loss of free thiol groups on cysteine residues is a form of protein oxidation associated with aging and cataractogenesis. Hains and Truscott measured the relative abundance of oxidized and disulfide bonded cysteine in human lenses; however, DHA was not measured in that study (17). Similar to Hains and Truscott and Fan et al. (13) and as stated above, our data displayed three trends: (1) DHA/DHB abundance increased with ARNC but not age, (2) DHA/DHB abundance increased with both age and ARNC, and (3) DHA/DHB abundance had no relationship with aging and ARNC (17). Specifically, DHA levels in BFSP2 Cys326 (**Figure 4A**) and βA3-crystallin Cys170 (**Figure 4C**) were highest in ARNC lenses but no association with lens age was observed. A similar result for βA3-crystallin Cys170 cysteine oxidation was reported by Hains and Truscott (17). Another DHA site that displayed similar trends as those seen in previous work was βA3-crystallin Cys82 (**Figure 5C**). Hains and Truscott found that cysteine oxidation on βA3-crystallin Cys82 was highly related to aging and ARNC our results found DHA levels increased strongly with age (17) in senior ARNC donors but these results were less pronounced compared to the age-related trends observed. αA-crystallin Cys131 and Cys142 (**Supplementary Figure 3**) displayed age and ARNC related changes when measuring cysteine oxidation or DHA levels (17). These results suggest that DHA formation is correlated to cysteine oxidation. This finding is consistent with our previous study that disulfide bonded cysteine residues readily form DHA (39).

To understand the reactivity of cysteine oxidation during low GSH levels, Fan et al. (13) identified changes in disulfide bond levels. Proteins and residues associated with cysteine oxidation levels that increased with age and ARNC were βA3-crystallin Cys170 (**Figure 4C**), and βA4-crystallin Cys33 (**Figure 5D**) (13), which were also found to have increased DHA levels with age or ARNC. These results provide more evidence that DHA is correlated to cysteine oxidation. Fan et al. showed that cysteine oxidation was correlated to intermolecular disulfide crosslinks during the aging process and cataractogenesis as opposed to intramolecular disulfide crosslinks in younger lenses (13). We posit that DHA formation would similarly cause structural changes as the loss of intramolecular disulfide crosslinks. βB1-crystallin Cys79 (**Supplementary Figure 2**), and γC-crystallin Cys23 (**Figure 6B**) did not show increased levels with aging and ARNC with DHA abundance but did with oxidized cysteine levels (13), which may be due to the other nucleophiles in the lens. Interestingly, several sites that did not show cysteine oxidation levels change with age or ARNC did show a relationship with DHA levels and age or ARNC (13), such as αA-crystallin Cys131 and Cys142 (**Supplementary Figure 3**), βA3-crystallin Cys82 (**Figure 5C**), and βA4-crystallin Cys5 (**Figure 4B**). These results suggest that DHA abundance may be influenced by more than GSH levels and that DHA may be another barometer of cysteine oxidation.

DHA- and DHB-modified proteins were more likely to be found in the UIF fraction, as observed in 10 of the 13 quantified sites, regardless of the specific protein, native protein solubility, region of the protein, or trend identified. This was unexpected because non-modified protein and peptide counterparts were more likely to be in WSFs or USFs. This is exemplified by the shift in solubility of αA-crystallin from a typically water-soluble protein to a urea-soluble and urea-insoluble protein when DHA is present on residues Cys131 and Cys142 (**Supplementary Figure 4**). These data suggest that DHA and DHB may induce structural changes that cause protein insolubilization. A critique of this hypothesis arises from the fact that DHA can also be found in the other solubility fractions, albeit in much lower abundances. Furthermore, the likelihood of other protein modifications occurring increases with age, and these may also change the solubility of proteins. For example, previous research by Grey and Schey found that intact αA-crystallin could be found in younger lenses in all regions of the lens (40). However, intact αA-crystallin could not be found in the nucleus of older lenses and, instead, multiple subspecies of truncated αA-crystallin were found (40). Another hypothesis that may explain the low levels of soluble DHA containing proteins is that proteins with DHA/DHB could be targeted for degradation when soluble; however, once insoluble, they may escape proteostatic mechanisms.

Whether DHA and DHB formation is causing insolubilization and aggregation remains to be established. It is very likely that other modifications, known to accumulate with age, may be influencing protein structure in combination with DHA and the combined effects on protein structure will be difficult to determine. Alternatively, if DHA can induce the solubility changes seen in this study, it is possible that protein precipitation or aggregation occurs when DHA or DHB forms on multiple sites of the same protein (41). Further testing is necessary to determine what effect DHA or DHB has on protein structures. Some of the factors that may influence the impact of DHA/DHB formation at each identified site are accessibility of the site, the role of the site in protein-protein interactions or protein structure and flexibility, and the charge states and reactivity of nearby residues. Our data suggest that DHA formation affects protein solubility and may have some impact on protein rigidity. For example, DHA on BFSP2 residue Cys326 (**Figure 4A**) showed no age-related trend but an ARNC trend whereas BFSP2 Cys255 (**Figure 5A**) and Cys65 (**Figure 5B**) showed progressive increases with age. This difference suggests that regions within the same protein can have different effects on protein structure and solubility. If, as our data suggest, structural changes induced by DHA/DHB formation lead to solubility changes, then it is possible for some buried sites to be exposed only after significant protein modification. Thus, some modifications will accumulate with age while others appear only at advanced stages of denaturation. Evidence that may support this notion is that BFSP2 Cys326 is present in the coiled region of the protein whereas BFSP2 Cys65 and Cys255 are in the head and rod domains respectively. Cys326 is present in a region responsible for the overall shape of the dimer and flexibility of BFSP2 (42, 43). This buried residue is not as exposed as other regions and may only be accessible when BFSP2 is unfolded, which is more likely during the denaturing conditions of advanced age or cataractogenesis. In contrast, BFSP2 Cys65 and Cys255 are involved in the assembly of beaded filaments. An age-related trend may be more likely to occur for these more exposed residues.

Like BFSP2, βA4-crystallin containing DHA on Cys5 showed a strong relationship with ARNC (**Figure 4B**) while DHA-modified βA4-crystallin Cys33 showed a strong relationship with aging and ARNC (**Figure 5D**). βA4-crystallin Cys5 and Cys33 are located on subdomains within the protein that have distinct structural differences. Cys33 is buried within a Greek key motif; a secondary structure that allow regions within a protein to be highly compact and less vulnerable to stress (44). Previous studies found that when a mutation arises within a single Greek key motif the protein will then self-aggregate and precipitate (44). This information combined with the change in solubility (**Figure 5D**) suggests that DHA on Cys33 may cause structural deficits associated with function loss. When a mutation occurred in a region not associated with the Greek key motif, the protein remained more stable than its counterpart even if those changes could induce structural and functional deficits (44). That DHA on Cys33 increases with age suggests that, over time, misfolding or aggregation occurs, which is consistent with previous research (5). βA4-crystallin Cys5 (**Figure 4B**) is on the N-terminal arm of the protein, which is hypothesized to stabilize protein-protein interactions among complexes (45). Cys5 is a highly exposed residue, which shows high levels of DHA in ARNC similar to BFSP2 Cys326. When comparing the abundance of DHA on βA4-crystallin Cys5 to DHA-GSH crosslinks identified by Wang and Schey (25), high levels of DHA were found among senior cataractous lenses in this current study whereas DHA-GSH crosslinks where more likely to be identified among transparent senior lenses. DHA-GSH crosslinks were found in much higher abundances, which suggests that DHA formation and reaction with GSH may be one cause of age-related decline of GSH (46) and reaffirms the potential for DHA to impair lens transparency.

In contrast to the age and cataract specific trends described above, DHA levels at γC-crystallin residue Cys23 and βA3-crystallin T127 (seen in **Figure 6**) showed high abundance among younger lenses compared to senior or cataractous lenses. γC-crystallin Cys23 was not found to have a relationship between cysteine oxidation and aging or age-related diseases (17). We speculate that these residues are exposed on the protein surface, and upon, DHA/DHB formation, readily react with lens nucleophiles.

In summary, our results show that there is a relationship between DHA and DHB levels and age and ARNC. Many fewer sites with DHB were identified compared to DHA, which suggests that DHB containing peptides may need further enrichment for detection and quantification. Although DHA and DHB are known to contribute to high molecular weight crosslinks correlated to cataracts (33), it is unclear to what extent or how DHA or DHB may be involved in cataractogenesis since this modification is present throughout the aging process. However, our data suggest that for most of the proteins examined, DHA modifications shifted the protein solubility to the insoluble fraction, even for soluble crystallins. Further work is needed to determine the specific role of DHA on protein insolubilization, and our results suggest that this phenomenon may be protein and/or site-specific.

## Data availability statement

The data presented in the study are deposited in the ProteomeXchange Consortium via the PRIDE (47) partner repository, accession number PXD045734. The data can be accessed via URL: http://www.ebi.ac.uk/pride/archive/projects/PXD045734.

## Ethics statement

The studies involving humans were approved by Institutional Review Board, Vanderbilt University. The studies were conducted in accordance with the local legislation and institutional requirements. The human samples used in this study were acquired from another group as a gift and de-identified and others were obtained as de-identified samples from deceased donors. Written informed consent for participation was not required from the participants or the participants’ legal guardians/next of kin in accordance with the national legislation and institutional requirements.

## Author contributions

JP, ZW, PP, and KR performed experiments. JP and ZW analyzed data. JP, ZW, KR and KS designed experiments. JP and KS wrote manuscript. All authors contributed to the article and approved the submitted version.
