# Supplementary material for: Dehydroalanine and dehydrobutyrine in aging and cataractous lenses reveal site-specific consequences of spontaneous protein degradation
Source: Front Ophthalmol (Lausanne). 2023 Oct 26;3:1241001. doi: 10.3389/fopht.2023.1241001 (PMC11182102; doi:10.3389/fopht.2023.1241001)
Supplement: Supplementary file 1 [file DataSheet_1.pdf]

s

Supplementary Material

Dehydroalanine and Dehydrobutyrine in Aging and Cataractous Lenses Reveal Site-specific Consequences of Spontaneous Protein Degradation

Jessica Paredes, Zhen Wang, Purvi Patel, Kristie L. Rose, Kevin L. Schey*

*** Correspondence:** Corresponding Author: k.schey@vanderbilt.edu

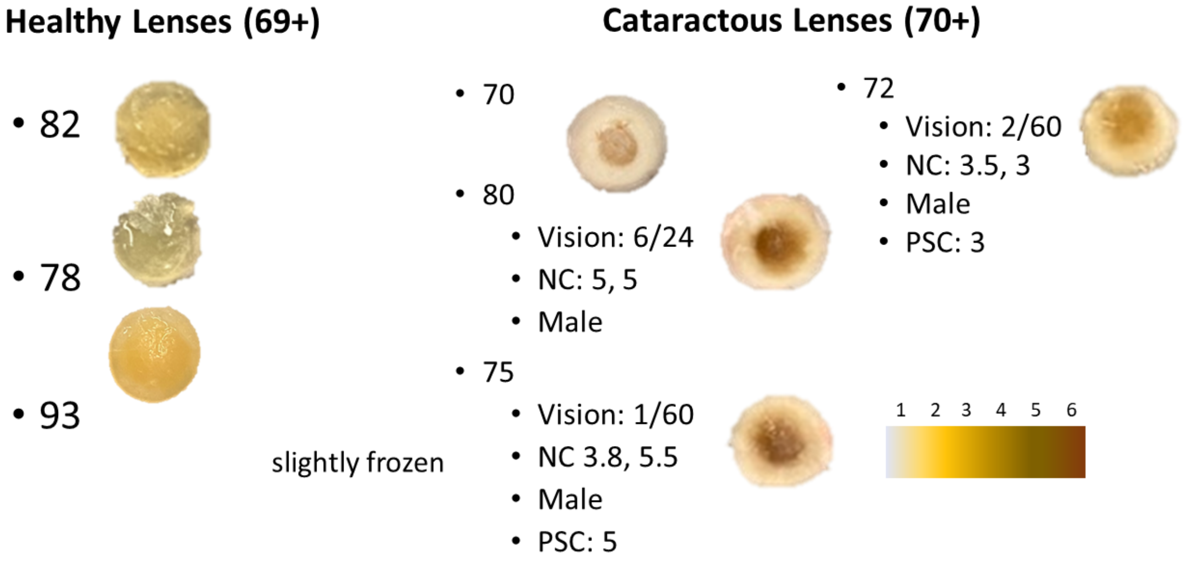

Supplementary Figure 1: Healthy lenses from donors (with ages depicted to the left of the picture of the lens) above 69 years old. Cataractous lenses from donors (with ages depicted to the left of the picture of the lens) above 70 years old. Visual acuity of the donor prior to donation if known is described. NC, nuclear color of a cataract based on the lens opacity classification system based on Chylack et al in 1993 (58). PSC refers to posterior subcortical cataract and each number next to it is the grade assigned to the PSC. Grading was collected by two different doctors to verify the nuclear color. The lens opacity classification system describes the severity and type of cataract from 1.0 (lens is more white than yellow), 2.0 (lens is more yellow than white), 3.0 (lens is lemon yellow), 4.0 (lens is gold), 5.0 (lens is bronze), and 6.0 (reddish bronze) after pupil dilation and inspected with a slitlamp, constant illumination, and no neutral filter. The slitlamp had a 5V light setting at a 45-degree angle, a slit height that overlap with the pupil, and slit width of 0.2 mm.

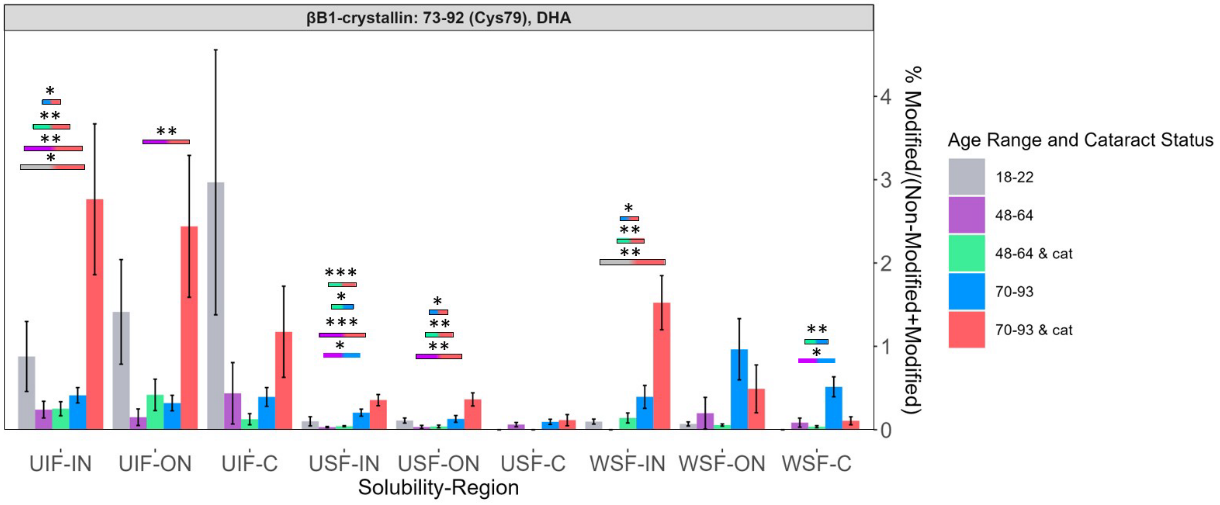

Supplementary Figure 2: Relative levels of DHA are shown for βB1-crystallin Cys79. Boxes above each result are colored to show which groups were statistically significant corresponding to the legend and represent a p-value less than 0.05. Outlined boxes represent comparisons where at least one lens group has cataracts; boxes without outlines represent comparisons where neither lens groups have cataracts. * represents a p-value less than 0.05; ** represents a p-value less than 0.01; and *** represents a p-value less than 0.001.

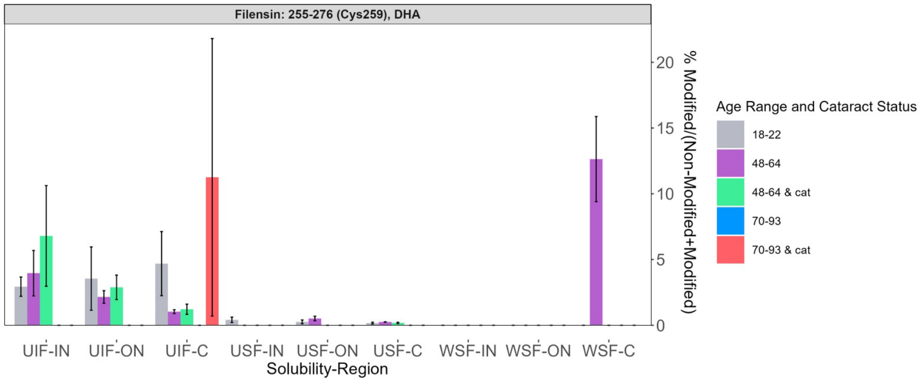

Supplementary Figure 3: Relative levels of DHA are shown for filensin (BFSP1) Cys259.

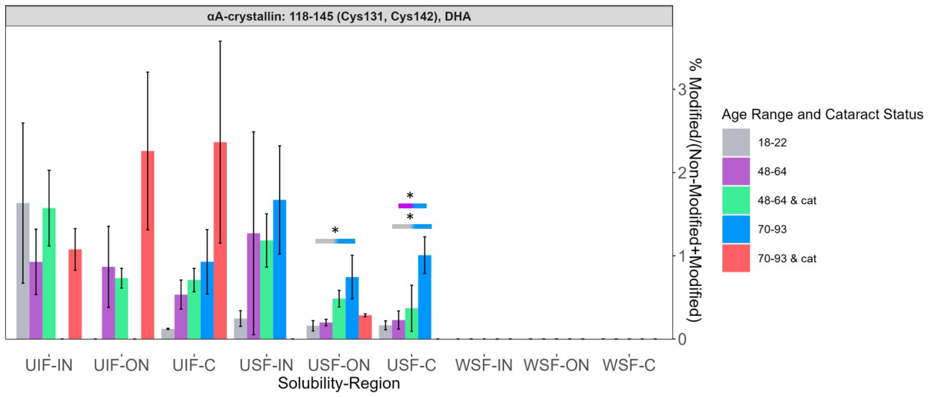

Supplementary Figure 4: Relative levels of DHA are shown for αA-crystallin Cys131 and Cys142. * represents a p-value less than 0.05.

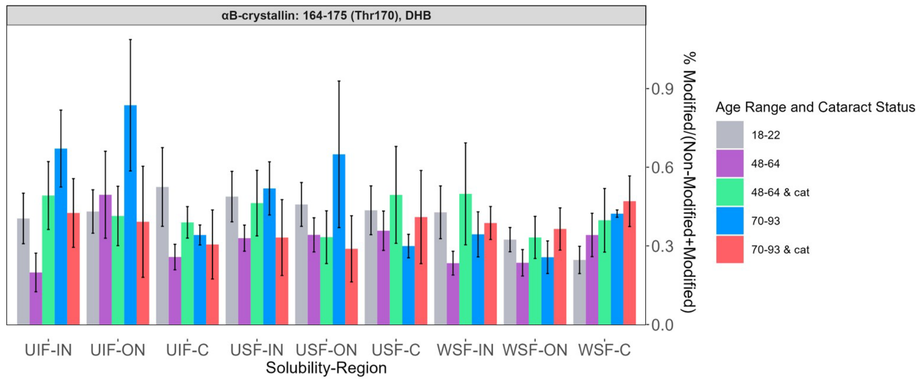

Supplementary Figure 5: Relative levels of DHB are shown for αB-crystallin Thr170.
